# Bilateral Breast Reconstruction with Abdominal Free Flaps: A Single Centre, Single Surgeon Retrospective Review of 55 Consecutive Patients

**DOI:** 10.1155/2016/6085624

**Published:** 2016-07-18

**Authors:** Peter McAllister, Isabel Teo, Kuen Chin, Boikanyo Makubate, David Alexander Munnoch

**Affiliations:** ^1^Department of Plastic and Reconstructive Surgery, Ninewells Hospital and Medical School, Dundee DD1 9SE, UK; ^2^Department of Public Health, Faculty of Medicine, University of Botswana, Gabarone, Botswana

## Abstract

Breast reconstruction using free tissue transfer is an increasingly utilised oncoplastic procedure. The aim was to review all bilateral breast reconstructions using abdominal free flaps by a single surgeon over an 11-year period (2003–2014). A retrospective review was performed on all patients who underwent bilateral breast reconstruction using abdominal free flaps between 2003 and 2014 by the senior author (DAM). Data analysed included patient demographics, indication for reconstruction, surgical details, and complications. Fifty-five female patients (mean 48.6 years [24–71 years]) had bilateral breast reconstruction. The majority (41, 74.5%) underwent immediate reconstruction and DIEP flaps were utilised on 41 (74.5%) occasions. Major surgical complications occurred in 6 (10.9%) patients, all of which were postoperative vascular compromise of the flap. Failure to salvage the reconstruction occurred on 3 (5.5%) occasions resulting in a total flap failure rate of 2.7%. Obesity (>30 kg/m^2^) and age > 60 years were shown to have a statistically increased risk of developing postoperative complications (*P* < 0.05). Our experience demonstrates that abdominal free flaps for bilateral breast reconstruction fares well, with a flap failure rate of 2.7%. Increased body mass index and patient age (>60 years) were associated with higher complication rates.

## 1. Introduction

In recent years, the number of bilateral breast reconstruction procedures has been increasing. This is a reflection of advancements in breast cancer screening and diagnosis supplemented by a desire for prophylactic surgery on the contralateral side. Positive genetic markers for BRCA confer an 85% lifetime risk of breast cancer, making this group of patients highly suitable for prophylactic mastectomies and reconstruction [1–4].

The options for reconstructing a breast mound following mastectomy include implants with or without autologous tissue flaps and can be performed at the time of the mastectomy (immediate) or at a later stage (delayed). Expanders and implants are a popular choice of breast reconstruction; however, the aesthetic outcomes are known to deteriorate with time, particularly in the context of radiation therapy [5–8]. The 2011 National Mastectomy and Breast Reconstruction audit, in which 8,159 women were sent patient satisfaction questionnaires, identified superior reported outcomes in patients who had received autologous tissue reconstruction compared to those with implant only reconstruction.

There are various choices of flaps for both pedicled and free flap breast reconstruction. Common pedicled options include the latissimus dorsi flap and the pedicled transverse rectus abdominis myocutaneous (TRAM) flap. The former is a reliable and robust flap but often requires an implant to augment the breast mound. The pedicled TRAM has traditionally been the workhorse for autologous breast reconstruction but is associated with higher rates of fat necrosis and abdominal wall hernia compared to free abdominal flap surgery. The free TRAM (where a small amount of muscle between the perforators and main pedicle is taken with flap harvest) has a role in certain patients but is known to have poorer outcomes compared to the muscle-preserving DIEP perforator technique which is associated with minimal functional loss and less postoperative pain [5–8]. The superficial inferior epigastric artery (SIEA) flap supplies less skin and fat abdominal tissue than the DIEP flap and is not always present [9].

The DIEP flap is currently widely regarded as the gold standard for breast reconstruction. It preserves the underlying musculofascial layer which reduces postoperative pain, duration of hospital admission, and long term donor site morbidity [10–12].

The superior cosmetic outcomes and acceptable morbidity rates of abdominal free flaps for unilateral breast reconstruction have been well published; however, the literature on bilateral procedures is limited.

The aim of this study was to review our experience with bilateral abdominal free flap reconstruction, analyse patient demographic and surgical data, and identify potential risk factors which may predispose to postoperative complications.

## 2. Materials and Methods

A retrospective case note review was performed of patients who had undergone free abdominal tissue transfer for bilateral breast reconstruction between August 2003 and February 2014. The list of patients was identified through a contemporaneous logbook of the senior author.

Patient demographic information including age, medical history, tobacco use, weight (body mass index (BMI) (kg/m^2^)), and family and genetic history for breast cancer were recorded.

Surgical data included indication for breast resection (therapeutic or prophylactic), reconstructive timing, type of abdominal flap harvested, ischaemic time, recipient vessels, flap size, and hospital stay. Adjuvant radiotherapy and/or chemotherapy were also documented.

Data on complications of surgery were reviewed. Complications were classified as early (occurring within seven days of initial surgery) or late (occurring seven days after initial surgery).

The unit of investigation for data relating to the flap was the number of flaps (percentages). All other data were described as number of patients (percentages). Chi-squared tests for trend were used to compare 2 (two) groups. Chi-squared *P* values of less than 0.05 were considered significant. All statistical analyses were performed with IBM SPSS Statistics Version 22.0. (IBM Corp. (2013)) [13].

## 3. Surgical Technique

The majority of patients underwent standard DIEP flap reconstruction but the following may differ between units.

### 3.1. Preoperative

All patients are assessed at a preoperative anaesthetic assessment clinic approximately one month before surgery. They are admitted one day before surgery and seen by the senior author. A handheld Doppler probe is used to map potential perforators. We do not employ the routine use of CT-angiography. Preoperative low molecular weight heparin is not given and tamoxifen is not stopped prior to surgery.

### 3.2. Anaesthetic Considerations

Following intubation, all patients receive an arterial line and urinary catheter to monitor cardiovascular status. The abdominal flap edges are infiltrated with local anaesthetic and adrenaline. The mixture used is 60 mLs of 0.9% saline with 40 mLs of 0.5% levobupivicaine and 1 mL of 1 : 1000 adrenaline. Pneumatic compression stockings are utilised and patients receive intravenous Flucloxacillin on induction (clindamycin for patients with a Penicillin allergy).

### 3.3. Surgery

The patient is prepped with Betadine and a minimum of two surgeons operate simultaneously, the senior author and a trainee. In immediate cases, the contralateral flap is raised as the breast surgeon performs the mastectomy, with or without axillary node clearance. The mastectomies performed are commonly skin sparing. The superficial inferior epigastric vessels, if present, are routinely harvested prior to identifying DIEP perforators. Often two perforators are harvested and motor nerves preserved. The decision to use lateral or medial row perforators is made intraoperatively depending on location and size. The recipient vessels of choice, usually the internal mammary artery and vein, are exposed by removing the third intercostal cartilage. Anastomoses are performed end to end with 9/0 Ethilon. Venous couplers and intraoperative anticoagulants are not used. Two drains are placed in each breast pocket and one drain is placed on each side of the abdomen.

### 3.4. Postoperative Care

All patients return to a single room on the high dependency unit (HDU) and have hourly clinical flap observations overnight. All patients receive 24 hours of intravenous Flucloxacillin and 2500 units of low molecular weight heparin (LMWH) twice daily. Progressive mobilisation is encouraged and drains are kept until the patient is mobile with less than 30 mLs in 24 hours.

## 4. Results

The medical records of 55 consecutive patients (mean age 48 years, range 24–71 years) undergoing simultaneous bilateral breast reconstruction surgery with abdominal autologous free flaps (*n* = 110) were reviewed. The average follow-up to the time of this study was 46.1 (4–130) months. Patient demographics are provided in Table 1.

The majority of patients (41; 75%) underwent immediate reconstruction, 8 (15%) had a delayed reconstruction, and 6 (11%) had an immediate reconstruction unilaterally and a delayed reconstruction contralaterally. Surgical data are illustrated in Table 2.

Therapeutic surgery for unilateral or bilateral disease accounted for 45 (81.8%) patient breast resections. Invasive ductal carcinoma (22; 54%) and ductal carcinoma in situ (DCIS) (6; 15%) were most frequently encountered.

In this series, 12 (22%) patients were BRCA positive, and 12 (22%) had a strong family history of breast cancer (disease affecting a first-degree female relative) but were BRCA negative and 31 (56%) had no known familial or genetic risk factors.

Of the BRCA positive patients, 7 (13%) underwent unilateral therapeutic and contralateral prophylactic resection and 5 (9%) had bilateral prophylactic (risk reducing) mastectomies. Of the patients with a strong familial history (BRCA negative), 7 (13%) underwent unilateral therapeutic and contralateral prophylactic surgery and 5 (9%) patients requested bilateral prophylactic resection of breast tissue in the absence of identifiable pathology (Table 1).

Complications were classified as early (occurring within seven days of initial surgery) or late (occurring more than seven days after initial surgery). Forty-two patients (76%) experienced one or more early or late complications of flap, donor site, or both. All early complications (*n* = 6) were vascular problems of the free flap and all of these underwent immediate surgical exploration (Table 3). Three flaps were salvaged and three eventually failed and required surgical debridement. The most common late complication in the breast/flap site was native breast skin necrosis 12 (11%) and the most common late complication of the donor site was wound dehiscence 23 (41%). This is shown in Table 4.

We performed analysis of risk factors to identify potential predisposing features that may increase risk of complications. Specifically we analysed smoking, raised BMI, previous abdominal surgery, age, flap weight, and ischaemic time and adjuvant radiation therapy.

There were 15 patients who were active or ex-smokers, and this risk factor was not found to statistically increase the risk of overall complications (*P* = 0.118).

Obese patients were found to have increased risk of complications. Of 19 (35%) patients with a BMI greater than or equal to 30 kg/m^2^, 15 (78.9%) (*P* = 0.019) experienced flap complications, donor site complications, or both. This is in contrast to the group of patients with a BMI < 30 kg/m^2^ where flap/and or complication rate was 19 (53%).

Increasing age was associated with increased complications. The majority of patients (47; 85%) were under 60 years of age, and, of eight patients older than 60 years, 6 (75%) experienced flap and/or abdominal complications postoperatively. There was a statistically increased risk of abdominal wound dehiscence (2/8, 25%,  *P* = 0.02) and native breast skin necrosis (2/8, 25%, *P* = 0.039) in this >60-year-old subgroup.

The weight of the flaps did not appear to affect complication rates and ranged in size from 280 g to 1200 g. Of 21 patients (38%) who had at least one flap exceeding 700 g in weight, 15 (71%, *P* = 0.078) experienced flap and/or abdominal complications of surgery but this was not regarded statistically significant.

In addition, flap ischaemic time did not appear to statistically affect complication rates. Ischaemic time, greater than 90 minutes for one or both sides, occurred in 17 (31%) patients, of which 12 (71%) (*P* = 0.143) encountered early or late flap complications.

Previous abdominal surgery did not have an effect on complication rates. Previous abdominal surgery was documented on 12 (22%) occasions and 4 (33%, *P* = 0.388) of these developed a degree of wound dehiscence postoperatively.

Radiation therapy was utilised in 29 (53%) patients (Table 2). Chest wall radiotherapy predating breast reconstruction was used in 15 (27%) patients. Time delay following radiotherapy to reconstruction varied from six months to 16 years and two (4%) patients experienced vascular complications of the venous anastomoses, one intraoperatively and one postoperatively. On surgical exploration, one was reanastomosed to the internal mammary vessels with no complications. One flap was anastomosed to the lateral chest wall vessels due to a concern regarding the original recipient chest wall vessel; this flap underwent partial flap failure.

Adjuvant radiotherapy after reconstruction was used in 14 (25%) patients with a mean duration of nine weeks following surgery. One patient experienced a significant loss of breast volume as a consequence of radiation treatment and underwent lipofilling to restore breast bulk. There were no other complications reported in this patient group.

Flap complications, abdominal complications, and associated risk factors are demonstrated in Tables 5 and 6, respectively. No patient encountered systemic complications as a consequence of reconstructive breast surgery. There were no pulmonary emboli, deep vein thrombosis, or sepsis events.

At the most recent follow-up, 30 (55%) patients had underwent further elective surgery, with nipple tattoo or nipple reconstruction accounting for half of these procedures. Three (5.4%) patients had been diagnosed with recurrent or metastatic disease and one (1.8%) was recently deceased secondary to fulminant heart failure.

## 5. Discussion

**Figure 1:**
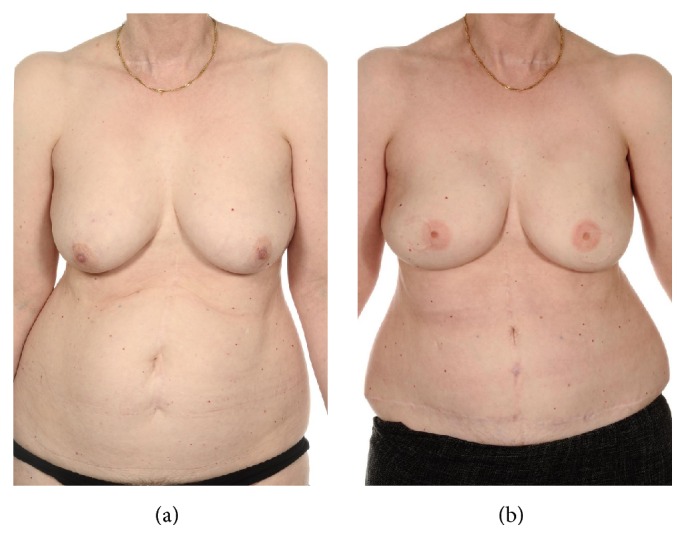
((a) Preoperative; (b) postoperative breast and nipple reconstruction) 56-year-old with invasive ductal carcinoma underwent bilateral mastectomy and immediate bilateral DIEP reconstruction and delayed nipple reconstruction (consent obtained to use photographs).

The use of abdominal donor perforator free flaps for breast reconstruction is widely advocated, with the DIEP flap heralded as the gold standard for breast reconstruction. This technique preserves the abdominal musculofascial system and is recognised as providing a favourable aesthetic outcome with low donor site morbidity (Figure 1) [14, 20]. The use of the DIEP flap in unilateral breast reconstruction is accepted and known to provide excellent cosmetic results and an acceptable morbidity profile. However, the literature on bilateral DIEP procedures is limited. A literature search identified only six similar publications reporting a bilateral DIEP series (Table 7). As illustrated, our early complications associated with flap vascularity (11%) (0%–12.5%), partial flap failure (1.8%) (0%–3.6%), and total flap failure (5.4%) (0%–9.6%) are comparable with previous studies [14–19].

In our study, 42 patients (76%) experienced one or more early or late complications of flap, donor site, or both.

All early complications (*n* = 6) involved vascular compromise of the flap and all of these required immediate surgical intervention; five of these underwent reanastomosis and three were salvaged. One patient's anastomosis was found to have active flow during reexploration and reanastomosis was not carried out. Anastomotic venous complication occurred in all but 1 case. Of the failed flaps, two were venous and one was arterial in origin and all of these had a failed reanastomosis (Table 3). Venous anastomotic compromise occurred more commonly than those of arterial origin, a finding in keeping with similar literature [21, 22].

Factor V Leiden (FVL) genetic mutation was subsequently identified in one patient with total flap failure following haematological investigation. Reports of free flap thrombosis in this group of patients leading to total or partial flap loss have previously been published [23, 24]. FVL is the most common inherited cause of hypercoagulability and leads to resistance of activated protein C leading to an increased risk of thrombotic events [25]. The prevalence of FVL is between 2% and 10% in the Caucasian population [26]. A personal or family history of unexplained thrombosis should raise the suspicion of FLV. It has been suggested that a preoperative thrombophilia screen in high risk patients should be carried out and consideration should be given for pedicled rather than free flap reconstruction in this group of patients [25, 26].

Our first total flap failure (2005) precluded routine haematological investigation for thrombophilia which is now routine following vascular compromise of the flap. However, the substantial size of flap (1200 g) may have contributed to its vascular compromise and failure. A negative thrombophilia screen was noted in the third patient and no obvious cause of flap failure has subsequently been identified.

In similar works, late complications are inconsistently defined and variably reported which makes comparison difficult [1]. Our late complication rate may be attributed to frequent outpatient follow-up over a long period of time (mean 46.1 months). Previous comparable studies had a shorter follow-up period (mean 14.6–32.1 months) [14, 16, 18]. Another possible contributing factor which may account for the late complications observed is the higher mean BMI (28.1 kg/m^2^) and/or increased active or recent tobacco usage (27.2%) in our cohort of patients compared with analogous literature on bilateral breast reconstruction [14–19].

Active or recent tobacco use was associated with late complications of the flap, including native breast skin necrosis, fat necrosis, and flap failure.

Guerra et al. [14] did not find a significant increased risk of fat necrosis or flap loss in smokers but did report an increased risk of breast wound dehiscence in this group of patients. Gill et al. [27] reviewed 758 unilateral and bilateral DIEP flaps and identified an increased risk of donor site complications in patients using tobacco. In contrast, our study donor site complication rates were not statistically significant among the smoking patient cohort.

Obesity is associated with an almost 12-fold increased risk of postoperative complications after breast surgery. Obese women often have bigger breasts which require a larger abdominal donor graft to be harvested. Further complications include delayed wound healing and longer postoperative recovery periods [28, 29].

The link between patient obesity and flap size is reflected in the observed rate of flap and/or abdominal complications in flaps exceeding 700 grams which was increased although not statistically significant (*P* = 0.078). Interestingly, a similar correlation between BMI, increased flap size, and overall complication rates was not identified by previous research on bilateral breast reconstruction [14].

**Figure 2:**
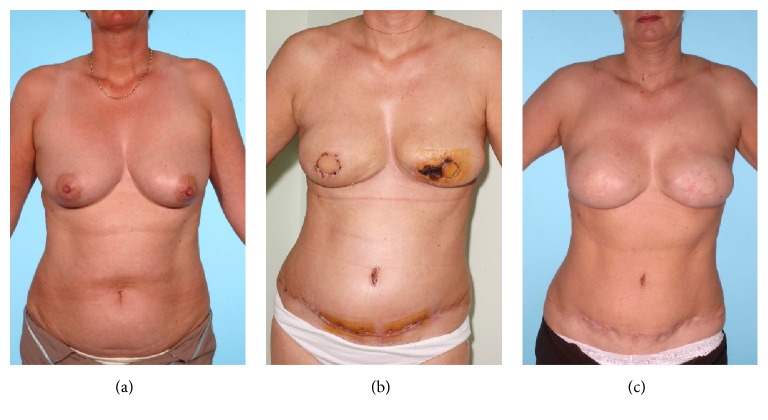
((a) Preoperative; (b) 4 weeks after reconstruction; and (c) 6 months after reconstruction) 42-year-old smoker with family history of breast and ovarian cancer (BRACA negative) underwent risk reducing bilateral mastectomy and immediate DIEP reconstruction and delayed nipple reconstruction. Note native breast necrosis and abdominal wound dehiscence (consent obtained to use photographs).

The most common late complications of flap and donor site were native breast skin necrosis (12/110; 11%) and abdominal wound dehiscence (13/55; 24%), respectively (Figure 2). Patients aged 60 years or older were noted to have a statistically increased risk of developing these postoperative complications (Tables 5 and 6). Concomitant disease and decreased physical function are associated with higher levels of surgical morbidity in elderly patients undergoing breast surgery [30] and this is endorsed by the findings of this study.

Complications associated with radiotherapy in the context of autologous breast reconstruction are a topic of debate [31–35].

In our series of bilateral free tissue reconstructions, two patients with preoperative chest wall irradiation experienced vascular problems of the flap, one salvaged with no complications and one salvaged with partial flap failure. Overall complication rate in this patient cohort was not statistically significant. This finding is in keeping with Berry et al. [31] who on multivariate analysis identified no statistical difference in rates of complications between patients receiving preoperative radiation therapy and those who did not.

In those patients undergoing postoperative radiotherapy, only one patient was observed to have a reduction in breast volume following adjuvant therapy. No other complications were observed. This is similar to work by Chatterjee and coworkers [32] who concluded that postoperative radiotherapy did not significantly affect breast volume after reconstruction.

Overall complication rate in patients who had chest wall irradiation after autologous breast reconstruction in our series was not significant, a finding consistent with some literatures [34] and contradictory to others [33, 35].

**Figure 3:**
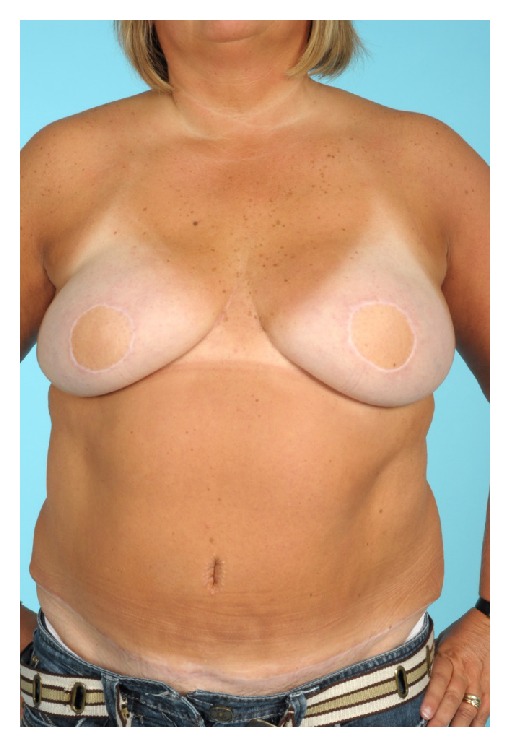
43-year-old immediate bilateral DIEP reconstruction for DCIS. Photograph taken after recent holiday abroad. Note skin paddle pigmentation in response to abdominal sun exposure (consent obtained to use photographs).

The behaviour of autologous transplanted tissue and its genetic tendency to mimic donor site are illustrated in Figure 3. The phenomenon of unexposed reconstructed breast skin paddles changing pigmentation in response to abdominal sun exposure is not described in the literature reviewed [5, 11, 12, 14–20].

Abdominal free flaps are unique as the use of this flap in a unilateral setting precludes future use for the contralateral side. In a recent publication, Wormald et al. [1] performed a systematic review comparing the risks of unilateral versus bilateral DIEP reconstructions and observed bilateral DIEP reconstructions to be associated with a significantly higher risk of total flap failure (RR 3.31, *P* = 0.003) and breast seroma (RR 7.15, *P* = 0.03), while other outcomes were comparable. At present, in non-BRCA patients with unilateral malignancy, bilateral mastectomies are not advocated as the risks of developing breast cancer in the contralateral side are comparable to the general female population. At our centre, all patients with a positive family history are sent for genetic testing, and lifetime risk estimates are provided for the patient to make an informed decision on the appropriateness of a contralateral prophylactic procedure. In this series, 12 (21.8%) patients were BRCA positive and 12 (22%) had a strong family history of breast cancer. Of these, 14 (26%) had identified breast pathology and underwent therapeutic surgery and 10 (18%) opted to have bilateral prophylactic (risk reducing) mastectomies.

Thirty-one (56%) patients with unilateral disease and no identifiable genetic or familial predisposition underwent bilateral mastectomy and reconstructive surgery. The decision to offer contralateral prophylactic mastectomy in a non-BRCA patient with no family history is controversial. However, in certain patients where there is a specific request for contralateral risk reducing procedure, a joint decision can be made with the breast surgeons, geneticist, oncologist, and plastic surgeon.

Limitations of the study must be considered. Retrospective case series studies are at risk of selection and reporting bias which reduce the reliability of results obtained. Prospective observational cohort studies to review objective and patient associated outcomes and reduce bias are considered preferable [1]. In addition, the relatively small size ensures that the study is insufficiently powered to accurately detect and predict uncommon but serious outcomes such as total flap failure. Moreover, utilising “the flap” as the unit of investigation for flap-associated data as opposed to “the patient” underestimates the clinical adverse outcomes. It has been suggested that using “the flap” as a data unit in bilateral breast reconstruction studies does not take into account “the patient” who ultimately has suffered from the complication that has arisen [1]. Finally undefined parameters pertaining to observer descriptions of complication presentations are a limitation of this study and similar studies.

## 6. Conclusion

Our experience demonstrates that abdominal free flaps for bilateral breast reconstruction fare well, with a flap failure rate of 2.7%. On analysis, smoking status, radiotherapy, flap weight, previous abdominal surgery, and ischaemic time were not associated with increased postoperative risks. We have found that an increased BMI and increasing age (>60 years) were associated with higher complication rates. This is useful information to help clinicians in decision making and counselling of future patients. Abdominal free flap surgery for bilateral breast reconstruction will continue to rise and the increasing popularity is a reflection of the natural evolution of breast reconstruction techniques to obtain maximal patient satisfaction and outcome.

## Figures and Tables

**Table 1:** Demographics of patients undergoing bilateral breast reconstruction.

*Number of patients/flaps*	*55/110*
Average age (years)	48.6 (24–71)
Average BMI (kg/m^2^)	28.1 (21–37)
Patients with medical comorbidities^*∗*^ (%)	16 (29)
Previous abdominal surgery (%)	25 (45)
Smokers (%)	7 (13)
Ex-smokers (%)	8 (15)
No family history of breast cancer^*∗∗*^ and BRCA negative with disease	31 (56)
Family history of breast cancer (BRCA negative) (%)	12 (22)
Family history of breast cancer with known disease (%)	7 (13)
Family history of breast cancer with no known disease (%)	5 (9.1)
BRACA gene positive (%)	12 (22)
BRACA positive with known disease	7 (13)
BRACA positive with no known disease	5 (9.1)

^*∗*^As per American Society of Anaesthesiology (ASA) II classification.

^*∗∗*^First-degree relative.

**Table 2:** Surgical data for patients undergoing bilateral breast reconstruction.

	*n* (%)
Therapeutic surgery (known disease)	45 (82)
Prophylactic surgery (no known disease)	10 (18)
Immediate reconstruction	41 (75)
After prophylactic mastectomy	8 (15)
After therapeutic mastectomy	33 (60)
Delayed reconstruction, mean delay (years)	8 (15)
After prophylactic mastectomy	2 (3.6)
After therapeutic mastectomy	6 (11)
Immediate/delayed	6 (11)
Abdominal donor	55 (100)
DIEP	41 (75)
TRAM	7 (13)
SIEA	1 (1.8)
Combination	6 (11)
Average ischaemic time (mins)	68.57
Recipient vein	
Internal mammary	100 (90)
Serratus	4 (3.6)
Thoracodorsal	2 (1.8)
Additional perforator	4 (3.6)
Recipient artery	
Internal mammary	106 (96)
Serratus	2 (1.8)
Thoracodorsal	2 (1.8)
Average flap size (g)	692.25
Peri/post-op blood transfusion	8 (15)
Average hospital stay (days)	7.5
Average follow-up (months)	46.1
Adjuvant chemotherapy only	8 (15)
Adjuvant radiotherapy only	6 (11)
Adjuvant chemoradiotherapy	23 (42)

**Table 3:** Early complications requiring unplanned surgical intervention.

	Number (%)
*Total*	*6/110*
Vascular complications	6 (5.4)
Anastomotic venous complication	5
Anastomotic arterial complication	1
Partial flap failure	1
Total flap failure	3 (2.7)

**Table 4:** Late complications.

	Number (%)
Breast/flap complications	18/110 (16.4)
Native breast necrosis	12 (11)
Fat necrosis	4 (3.6)
Seroma	1 (0.9)
Haematoma	1
Donor site complications	23/55 (42)
Dehiscence	13 (24)
Seroma	5 (9.1)
Hernia	1 (1.8)
Hypertrophic scarring	2 (3.6)
Lymphedema	1 (1.8)
Late donor site complications requiring surgery	1 (1.8)
Recurrence/metastasis	3 (5.4)

**Table 5:** Flap complications and associated risk factors.

	Native breast necrosis *n* (%)	*P* value	Fat necrosis *n* (%)	*P* value	Corrected vascular problem *n* (%)	*P* value	Partial/total flap failure *n* (%)	*P* value
Smoker/ex-smoker	7/15 (47)	0.774	2/15 (13)	1.000	0/3	0.250	2/15 (13)	1.000
Nonsmoker	5/40 (13)		2/40 (5)		3/3 (100)		2/40 (5)	
High BMI	8/19 (42)	0.388	1/19 (5.2)	0.625	1/19 (5.2)	1.000	2/19 (11)	1.000
Normal BMI	4/36 (11)		3/36 (8.3)		2/36 (5.6)		2/36 (5.6)	
Age > 60	2/8 (25)	0.039	0	0.125	0	0.250	0	0.125
Age < 60	10/47 (21)		4/47 (8.5)		3/47 (6.4)		4/47 (8.5)	
Flap > 700 g	4/21 (19)	0.388	2/21 (9.5)	1.000	2/21 (9.5)	1.000	2/21 (9.5)	1.000
Flap < 700 g	8/34 (24)		2/34 (5.9)		1/34 (2.9)		2/34 (5.9)	
Ischaemic time > 90 (mins)	5/17 (29)	0.774	1/17 (5.9)	0.625	2/17 (12)	1.000	2/17 (12)	1.000
Ischaemic time < 90 (mins)	7/38 (18)		3/38 (7.9)		1/38 (2.6)		2/38 (5.3)	

**Table 6:** Donor site (abdomen) complications and associated risk factors.

	Dehiscence *n* (%)	*P* value	Seroma *n* (%)	*P* value
Smoker/ex-smoker	5/15 (33)	0.581	1/15 (6.7)	0.375
Nonsmoker	8/40 (20)		4/40 (10)	
High BMI	7/19 (37)	1.000	4/19 (21)	0.375
Normal BMI	6/36 (17)		1/36 (2.8)	
Previous abdominal surgery	4/12 (33)	0.267	0	n/a
No previous abdominal surgery	9/43 (21)		0	n/a
Age > 60	2/8 (25)	0.022	1/8 (13)	0.375
Age < 60	11/47 (23)		4/47 (8.5)	
Flap > 700 g	8/21 (38)	0.581	2/21 (9.5)	1.000
Flap < 700 g	5/34 (15)		3/34 (8.8)	

**Table 7:** Complication rates in comparative bilateral breast reconstruction literature [1].

Study	*N*	TFF *n* ^*∗*^ (%)	PFF *n* ^*∗*^ (%)	VC *n* ^*∗*^ (%)	FN *n* ^*∗*^ (%)	H *n* ^*∗*^ (%)	AS *n* (%)	AH *n* (%)
Guerra et al. [14]	140	0	5 (3.6)	7 (5)	30 (21.4)	—	30 (21.4)	3 (2.1)
Scheer et al. [15]	32	2 (6.3)	1 (3.1)	4 (12.5)	12 (37.5)	3 (9.4)	0	4 (12.5)
Hofer et al. [16]	44	1 (2.3)	1 (2.3)	2 (4.6)	4 (9.1)	2 (4.6)	—	—
Drazan et al. [17]	55	0	—	0	2 (3.6)	4 (7.3)	2 (3.6)	—
Rao et al. [18]	114	11 (9.6)	—	—	—	—	—	—
Schaverien et al. [19]	10	0	0	0	1 (10)	—	0	0
Our study	55	3 (5.4)	1 (1.8)	6 (10.9)	4 (7.3)	1 (1.8)	5 (5.4)	0

TFF: total flap failure; PFF: partial flap failure; VC: vascular complications; FN: fat necrosis of flap; H: haematoma of flap; AS: abdominal seroma; and AH-abdominal hernia.

^*∗*^Unit of investigation is the “patient” and not the “flap” as per previous tables.
